# The spike protein of SARS-CoV-2 induces endothelial inflammation through integrin α5β1 and NF-κB signaling

**DOI:** 10.1016/j.jbc.2022.101695

**Published:** 2022-02-07

**Authors:** Juan Pablo Robles, Magdalena Zamora, Elva Adan-Castro, Lourdes Siqueiros-Marquez, Gonzalo Martinez de la Escalera, Carmen Clapp

**Affiliations:** Instituto de Neurobiología, Universidad Nacional Autónoma de México (UNAM), Querétaro, México

**Keywords:** spike protein, SARS-CoV-2, COVID-19, endothelial dysfunction, inflammation, integrin, NF-κB, permeability, endothelial cell, ACE2, ACE2, angiotensin-converting enzyme 2, BSA, bovine serum albumin, cDNA, complementary DNA, CD31, cluster of differentiation 31, CD45, cluster of differentiation 45, ConA, concanavalin A, COVID-19, coronavirus disease 2019, EC, endothelial cell, eNOS, endothelial nitric oxide synthase, FBS, fetal bovine serum, FVIII, factor VIII, GTPase, guanosine triphosphatase, HUVEC, human umbilical vein endothelial cell, ICAM1, intercellular cell adhesion molecule 1, IL-1β, interleukin 1 beta, IL-6, interleukin 6, IL-8, interleukin 8, IκB⍺, isoform ⍺ of IκB, mAb, monoclonal antibody, NO, nitric oxide, PBST, PBS with Tween-20, PFA, paraformaldehyde, RGD, arginine–glycine–aspartic acid, RT, room temperature, SARS-CoV-2, severe acute respiratory syndrome coronavirus 2, TEER, transendothelial electrical resistance, TF, tissue factor, TNF⍺, tumor necrosis factor alpha, VCAM1, vascular cell adhesion molecule 1

## Abstract

Vascular endothelial cells (ECs) form a critical interface between blood and tissues that maintains whole-body homeostasis. In COVID-19, disruption of the EC barrier results in edema, vascular inflammation, and coagulation, hallmarks of this severe disease. However, the mechanisms by which ECs are dysregulated in COVID-19 are unclear. Here, we show that the spike protein of SARS-CoV-2 alone activates the EC inflammatory phenotype in a manner dependent on integrin ⍺5β1 signaling. Incubation of human umbilical vein ECs with whole spike protein, its receptor-binding domain, or the integrin-binding tripeptide RGD induced the nuclear translocation of NF-κB and subsequent expression of leukocyte adhesion molecules (VCAM1 and ICAM1), coagulation factors (TF and FVIII), proinflammatory cytokines (TNFα, IL-1β, and IL-6), and ACE2, as well as the adhesion of peripheral blood leukocytes and hyperpermeability of the EC monolayer. In addition, inhibitors of integrin ⍺5β1 activation prevented these effects. Furthermore, these vascular effects occur *in vivo*, as revealed by the intravenous administration of spike, which increased expression of ICAM1, VCAM1, CD45, TNFα, IL-1β, and IL-6 in the lung, liver, kidney, and eye, and the intravitreal injection of spike, which disrupted the barrier function of retinal capillaries. We suggest that the spike protein, through its RGD motif in the receptor-binding domain, binds to integrin ⍺5β1 in ECs to activate the NF-κB target gene expression programs responsible for vascular leakage and leukocyte adhesion. These findings uncover a new direct action of SARS-CoV-2 on EC dysfunction and introduce integrin ⍺5β1 as a promising target for treating vascular inflammation in COVID-19.

Dysfunction of endothelial cells (ECs) has emerged as a major driver of coronavirus disease 2019 (COVID-19) (1, 2, 3). During resting state, ECs maintain their barrier function by limiting vasopermeability and preventing coagulation and inflammation. However, when activated in response to damage or infection, ECs produce chemoattractants, cytokines, and adhesion molecules, leading to vascular leakage, clot formation, inflammation, and leukocyte infiltration (4). Most people with severe COVID-19 die from acute respiratory distress syndrome, pulmonary edema, cytokine storm, multiple organ failure, and disseminated intravascular coagulation (5), all of which reflect EC dysfunction (1, 2, 3). Moreover, severe cases or deaths because of COVID-19 are associated with chronic endothelial damage from comorbidities, such as aging, obesity, hypertension, diabetes, and cardiovascular disorders (6, 7).

Potential mechanisms of vascular dysfunction in COVID-19 include EC death in response to severe acute respiratory syndrome coronavirus 2 (SARS-CoV-2) entry and replication (8), the binding of the spike protein of SARS-CoV-2 to the angiotensin-converting enzyme 2 (ACE2) receptor causing its downregulation and subsequent mitochondrial dysfunction (9), the activation of the proinflammatory kallikrein–bradykinin system, and the accumulation of proinflammatory and vasoconstrictor angiotensin II (2). In addition, the spike protein can trigger the expression of proinflammatory cytokines and chemokines (10), the production of toxic reactive oxygen species (11), and cell death (12). However, the underlying mechanisms of many of these effects remain unclear.

Alternative to ACE2, integrins may function as receptors mediating SARS-CoV-2 infection. Integrins are heterodimeric transmembrane cell adhesion molecules that exert various actions on hemostasis, inflammation, and angiogenesis. The spike protein contains an integrin-binding arginine–glycine–aspartic acid (RGD) motif exposed on the surface of the receptor-binding domain (13, 14) that binds to β1 integrins on pulmonary epithelial cells and monocytes (15). In particular, blockage of the binding of the spike protein to the integrin ⍺5β1 inhibits SARS-CoV-2 infection *in vitro* (16) and *in vivo* (17), demonstrating the therapeutic efficacy of targeting integrin ⍺5β1 in COVID-19. Furthermore, fibronectin ligation of integrin ⍺5β1 in ECs activates the transcriptional factor NF-κB responsible for the expression of proteins involved in inflammation and angiogenesis (18). These observations prompted us to investigate whether the binding of spike to the integrin α5β1 in ECs is sufficient to induce the endothelium proinflammatory phenotype.

## Results

### Spike stimulates leukocyte adhesion to ECs

**Figure 1:**
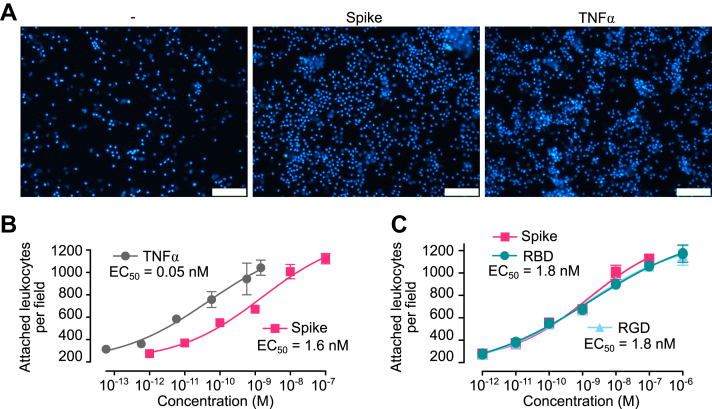
**Spike-induced leukocyte adhesion to EC.***A*, micrographs showing leukocytes adhered to HUVEC monolayers incubated in the absence (−) or the presence of spike (10 nM) or TNF⍺ (1 nM). The scale bar represents 120 μm. Dose–response stimulation of leukocyte adhesion to HUVECs in response to spike, TNF⍺. *B*, the receptor-binding domain of spike (RBD) or the RGD tripeptide. *C*, values are means ± SD, n = 9. Dose–response curves were fitted by least squares regression analysis (*r*^2^ > 0.93). EC, endothelial cell; HUVEC, human umbilical vein endothelial cell; RGD, arginine–glycine–aspartic acid; TNFα, tumor necrosis factor alpha.

Inflammatory changes can be assessed by the ability of ECs to attach leukocytes, a hallmark of the inflammatory process. Peripheral blood leukocytes were incubated for 1 h with human umbilical vein endothelial cells (HUVECs) pretreated for 16 h with spike, the receptor-binding domain of spike, the RGD tripeptide, or tumor necrosis factor alpha (TNF⍺) as a proinflammatory control (Fig. 1). Spike stimulated the adhesion of leukocytes to HUVECs in a dose-dependent manner with high potency (EC_50_ = 1.6 nM), and this stimulation paralleled the one induced by TNF⍺, a main inducer of EC proinflammatory changes (19) (Fig. 1, *A* and *B*). In addition, the receptor-binding domain of spike and the RGD tripeptide resulted in dose–response curves very similar to the one elicited by spike (EC_50_ = 1.8 nM) (Fig. 1*C*). These results show that spike alone activates the proinflammatory program in ECs and suggest that the RGD sequence located in the spike receptor–binding domain is responsible for this effect.

### Integrin α5β1 mediates spike-induced leukocyte adhesion to ECs

**Figure 2:**
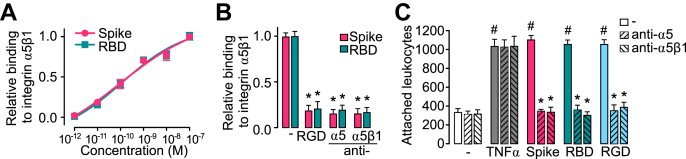
**Integrin α5β1 mediates spike-induced stimulation of leukocyte adhesion to EC.***A*, binding of different doses of spike or spike receptor–binding domain (RBD) to immobilized integrin α5β1 in an ELISA-based assay. Binding curves were fitted by least squares regression analysis (*r*^2^ > 0.97). *B*, effect of the RGD tripeptide, anti–integrin α5β1 antibodies or anti-α5 antibodies on the binding of spike or RBD to immobilized integrin α5β1. ∗*p* < 0.001 *versus* binding to spike or RBD (−) (two-way ANOVA, Šídák). *C*, leukocyte adhesion to a HUVEC monolayer in the absence (−) or the presence of 100 nM spike, RBD, RGD, or 1 nM TNF⍺, alone or together with antibodies against α5β1 integrin or the α5 integrin subunit. Values are means ± SD, n = 9, #*p* < 0.001 *versus* (−), ∗*p* < 0.001 *versus* the absence of antibodies (two-way ANOVA, Tukey test). EC, endothelial cell; HUVEC, human umbilical vein endothelial cell; RGD, arginine–glycine–aspartic acid; TNFα, tumor necrosis factor alpha.

RGD is the integrin-binding motif of several integrin ligands, including fibronectin, the main ligand of integrin α5β1. Fibronectin is upregulated during inflammation (20) and stimulates the EC inflammatory program (18). We used ELISA-based methods to confirm the binding of spike to integrin α5β1 (16). Plates were coated with integrin α5β1 and incubated with spike or the receptor-binding domain of spike in the absence or the presence of the RGD tripeptide or neutralizing antibodies against integrin α5β1 or the α5 integrin subunit. Spike and its receptor-binding domain bound α5β1 integrin with the same affinity (*K*_*d*_ = 200 pM) (Fig. 2*A*) and, as expected, the RGD tripeptide and the anti-α5β1 and anti-α5 antibodies prevented this binding (Fig. 2*B*). Because fibronectin ligation of integrin α5β1 induces the expression of adhesion molecules in ECs (18), we explored whether the binding to integrin α5β1 mediated the spike-induced stimulation of leukocyte attachment. The effect of anti-α5β1 or anti-α5 antibodies was tested against a dose (100 nM) of spike, spike receptor–binding domain, RGD tripeptide, or TNF⍺ (1 nM) having maximal stimulatory effect on leukocyte adhesion to HUVEC (Fig. 2*C*) to ensure that elicited actions were effectively taking place. Consistent with an integrin α5β1–dependent effect, both antibodies blocked leukocyte adhesion in response to spike, the spike receptor–binding domain, and the RGD tripeptide. The effect of TNFα was not modified, confirming that it is integrin independent (21).

### Spike-induced leukocyte adhesion to ECs is dependent on integrin α5β1–mediated NF-κB activation

**Figure 3:**
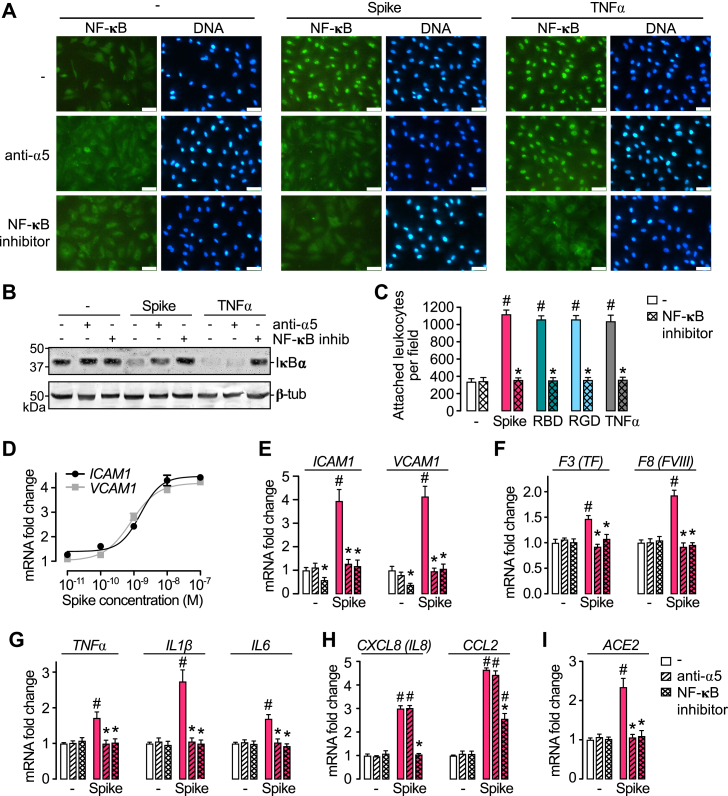
**NF-κB activation through integrin α5β1 mediates spike-induced EC leukocyte adhesion and inflammation.***A*, immunofluorescence detection of NF-κB (p65) in HUVEC incubated in the absence (−) or the presence of spike (100 nM) or TNF⍺ (1 nM) alone or in combination with anti-α5 antibodies or the NF-κB inhibitor, BAY 11-7085. The scale bar represents 50 μm. *B*, representative Western blot of IκB⍺ from HUVEC monolayers incubated without or with 100 nM spike or 1 nM TNF⍺ in the absence or the presence of anti-α5 antibodies or BAY 11-7085. β-tubulin is shown as control. *C*, leukocyte adhesion to HUVEC stimulated or not (−) by 1 nM TNFα or 100 nM spike, spike receptor–binding domain (RBD), or the RGD tripeptide alone or together with BAY 11-7085. *D*, dose–response stimulation of mRNA levels of intercellular cell adhesion molecule 1 (ICAM1) and vascular cell adhesion molecule 1 (VCAM1) in HUVEC incubated with different concentrations of spike. Dose–response curves were fitted by least square regression analysis (*r*^2^ > 0.9). HUVEC mRNA levels of adhesion molecules (ICAM1and VCAM1) (*E*); coagulation factors (F3/tissue factor and F8/coagulation factor VIII) (*F*); cytokines (TNF⍺, IL-1β, and IL-6) (*G*); chemokines (CXCL8/IL-8 and CCL2) (*H*); and ACE2 (*I*), after incubation with or without (−) 100 nM spike, in the absence or the presence of anti-α5 antibody or BAY 11-7085. Values are means ± SD, n = 9, #*p* < 0.001 *versus* the unstimulated control (−), ∗*p* < 0.001 *versus* the absence of BAY 11-7085 or anti-α5 antibodies (two-way ANOVA, Tukey test). ACE2, angiotensin-converting enzyme 2; CCL2, chemokine (C-C motif) ligand 2; CXCL8, chemokine (C-X-C motif) ligand 8; EC, endothelial cell; HUVEC, human umbilical vein endothelial cell; IL-1β, interleukin 1β; IL-6, interleukin 6; IκB⍺, isoform ⍺ of IκB; RGD, arginine–glycine–aspartic acid; TNF⍺, tumor necrosis factor alpha.

Because integrin α5β1 activates NF-κB in ECs to elicit inflammation (18), we asked whether the mechanism by which spike promotes leukocyte adhesion involved the activation of NF-κB. NF-κB is a transcriptional factor for numerous genes involved in inflammation that is held inactive in the cytoplasm by the interaction with proteins known as IκB. The phosphorylation/degradation of IκB is needed for the nuclear translocation of NF-κB and its binding to promoter and enhancer regions of target genes (22). The NF-κB cellular distribution was studied in HUVECs using fluorescence cytochemistry and a monoclonal antibody (mAb) against the p65 subunit of NF-κB. In the absence of treatment, p65 was homogeneously distributed throughout the cytoplasm of cells. Spike induced the accumulation of p65 in the cell nucleus, and this redistribution was like the one induced by TNFα (Fig. 3*A*). Anti-α5 antibodies blocked the spike-induced nuclear localization of p65 but not in response to TNFα; whereas, the NF-κB inhibitor BAY 11-7085 blocked the nuclear translocation of p65 induced by both spike and TNFα (Fig. 3*A*). Consistent with these observations, spike and TNFα induced the degradation of the isoform ⍺ of IκB (IκB⍺) in HUVEC, and anti-α5 antibodies prevented IκB⍺ degradation in response to spike but not in response to TNFα, whereas BAY 11-7085 prevented IκB⍺ degradation by both spike and TNFα (Fig. 3*B*). We conclude that spike activates NF-κB through its interaction with integrin α5β1.

To evaluate whether the spike-induced leukocyte adhesion to ECs is dependent on NF-κB activation, we tested the effect of BAY 11-7085 upon spike-induced, spike receptor–binding domain–induced, RGD tripeptide–induced, or TNF⍺-induced stimulation of leukocyte adhesion to HUVECs (Fig. 3*C*). Inhibition of NF-κB prevented the stimulation of leukocyte adhesion in response to all treatments (Fig. 3*C*). Furthermore, spike upregulated, in a dose-dependent manner, the mRNA levels of intercellular cell adhesion molecule 1 (ICAM1) and vascular cell adhesion molecule 1 (VCAM1), both of which mediate the firm adhesion of leukocytes to the apical surface of ECs (23) (Fig. 3*D*). The spike potency eliciting the expression of both adhesion molecules (EC_50_ = 1.58 nM for ICAM1; EC_50_ = 0.82 nM for VCAM1) was like the one (EC_50_ = 1.56 nM) promoting leukocyte attachment (Fig. 1*B*). Furthermore, the spike-induced increase of ICAM1 and VCAM1 expression was prevented by the inhibition of NF-κB and integrin α5 immunoneutralization (Fig. 3*E*). These findings show that the binding of spike to integrin α5β1 activates the NF-κB pathway in ECs responsible for leukocyte attachment.

### Spike induces the expression of proinflammatory and procoagulant genes in ECs in a manner dependent on α5β1 and NF-κB

Release of proinflammatory cytokines into the circulation (cytokine storm) and disseminated intravascular coagulation are markers of severe COVID-19 (1, 2, 3), and integrin α5β1 activation leads to NF-κB-dependent upregulation of proinflammatory cytokines, chemokines, and coagulation factors in ECs (18). Here, we show that spike increases the mRNA levels of coagulation factors (tissue factor [TF] and factor VIII [FVIII]) (Fig. 3*F*), proinflammatory cytokines (TNF⍺, interleukin 1 beta [IL-1β], and interleukin 6 [IL-6]) (Fig. 3*G*), and chemokines (chemokine [C-X-C motif] ligand 8/interleukin 8 [IL-8] and chemokine [C-C motif] ligand 2) (Fig. 3*H*) in HUVEC. Upregulation of coagulation factors and proinflammatory cytokines was prevented by integrin ⍺5 antibodies and the inhibitor of NF-κB, whereas the spike-induced expression of chemokines was only prevented by the inhibition of NF-κB and not integrin ⍺5 immunoneutralization (Fig. 3, *F*–*H*). These findings imply that spike activates NF-κB signaling pathways that are dependent and independent of α5β1 integrin to elicit the vascular proinflammatory and procoagulant state characteristic of severe COVID-19.

### Spike increases the expression of ACE2 in ECs through α5β1 and NF-κB

Spike binding to ACE2 is an alternative mechanism producing EC damage. Spike protein of SARS-CoV-2 downregulates ACE2 protein levels in ECs *via* the ubiquitin–proteasome system, which in turn leads to mitochondrial fragmentation, impaired endothelial nitric oxide (NO) synthase (eNOS) activity, dysregulated renin–angiotensin system, and severity of COVID-19 (9). Here, we show that spike upregulated (∼2.5-fold) the expression of ACE2 mRNA levels in HUVECs and that this effect was blocked by anti-α5 antibodies and the inhibitor of NF-κB (Fig. 3*I*). These findings imply that spike exerts dual actions on ACE2 levels in ECs depending on whether it binds to ACE2 or activates α5β1–NF-κB signaling pathways. Although ACE2 may be anti-inflammatory (24), its induction by spike could be part of a protective mechanism against EC injury. Alternatively, spike-induced ACE2 expression may worsen EC viral infection by providing more SARS-CoV-2 receptor.

### Spike stimulates the *in vivo* proinflammatory phenotype of ECs

**Figure 4:**
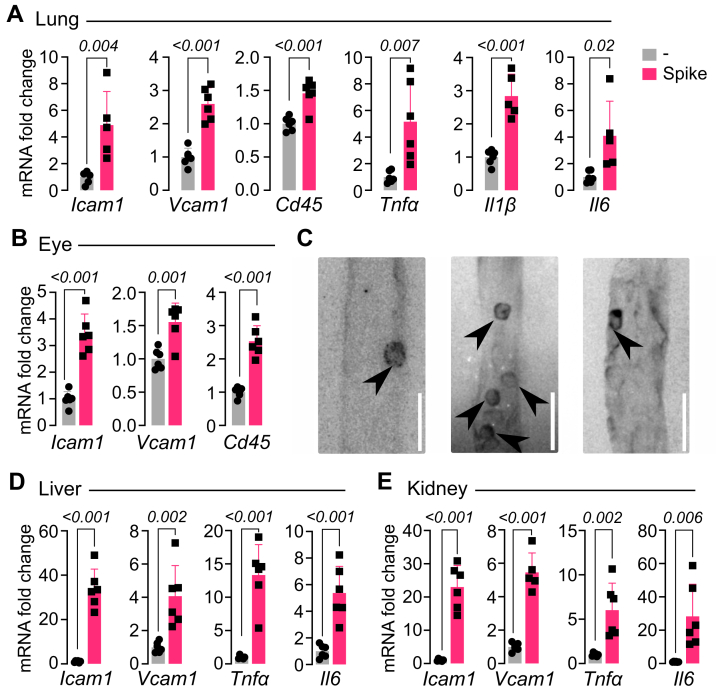
**Spike stimulates the *in vivo* proinflammatory phenotype of EC.** mRNA levels of adhesion molecules (ICAM1 and VCAM1), leukocyte marker (CD45), and cytokines (TNF⍺, IL-1β, and IL-6) in lung (*A*), eye (*B*), liver (*D*), and kidney (*E*) from mice after 2 h of being intravenously injected with 2.7 μg of spike. Values are means ± SD, n = 6. *p* Values are indicated in each case (unpaired *t* test). *C*, representative images of retinal flat mounts from spike-treated mice showing concanavalin A-labeled vessels containing numerous CD45-positive leukocytes (*arrowheads*). The scale bar represents 50 μm. EC, endothelial cell; ICAM1, intercellular cell adhesion molecule 1; IL-1β, interleukin 1β; IL-6, interleukin 6; TNF⍺, tumor necrosis factor alpha; VCAM1, vascular cell adhesion molecule 1.

To assess whether the spike protein alone could promote EC dysfunction *in vivo*, 2.7 μg of spike were injected i.v. to reach an estimated ∼10 nM concentration in serum, and after 2 h, mice were perfused and tissues (lung, liver, kidney, and eye) were collected to measure mRNA expression of ICAM1, VCAM1, leukocyte marker (cluster of differentiation 45 [CD45]), and proinflammatory cytokines (TNFα, IL-1β, and IL-6). The underlying rationale being that i.v. delivery and short-term (2 h) analysis in thoroughly perfused animals would reflect a direct effect of spike on EC mRNA expression of proinflammatory genes and leukocyte attachment to ECs in the various tissues. A single spike administration was enough to increase the expression levels of ICAM1 and VCAM1 in all tissues (Fig. 4), and this effect associated with enhanced levels of the leukocyte marker CD45 in the tissues measured (lung and eye) (Fig. 4, *A* and *B*). Leukocyte attachment to retinal vessels was evident for 4 h after i.v. injection of spike (Fig. 4*C*) but absent in vehicle-injected mice (not shown). Furthermore, spike increased the expression of TNF⍺, IL-1β, and IL-6 in the lung, liver, and kidney (Fig. 4). The similar observations among tissues are consistent with the ubiquitous nature of blood vessels and further suggest a direct action of spike on EC dysfunction *in vivo*. Systemic proinflammatory effects of spike have been reported previously (10, 25) using similar doses (1–8 μg) but longer times post-treatment (16 or 72 h) and different administration routes (intratracheal and intraperitoneal). These parameters likely reflect the action of spike on different cell types.

### Spike induces the hyperpermeability of EC monolayers *via* integrin α5β1

**Figure 5:**
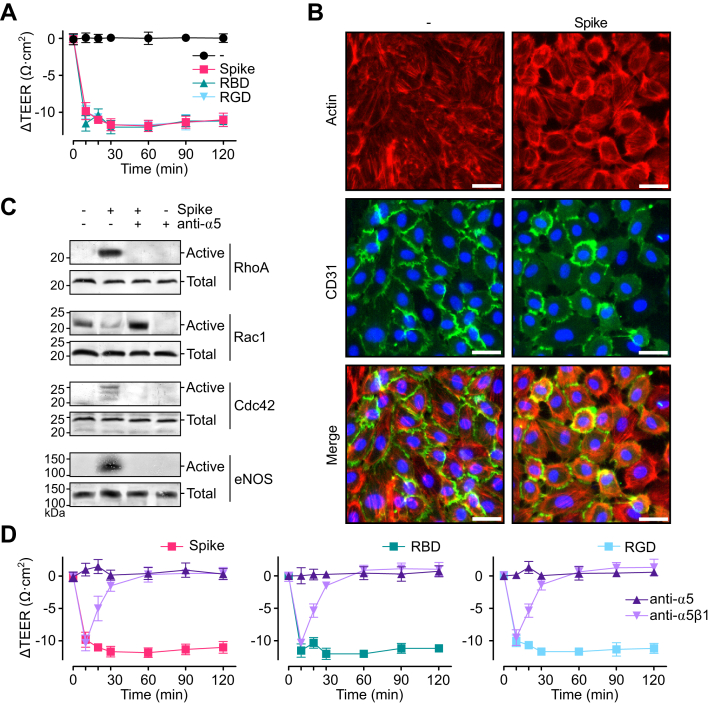
**Spike induces the hyperpermeability of EC monolayers *via* integrin α5β1.***A*, changes throughout time in the transendothelial electrical resistance (ΔTEER) of HUVEC monolayers incubated without (−) or with 100 nM spike, spike receptor–binding domain (RBD), or RGD tripeptide. *B*, representative fluorescence cytochemistry of a HUVEC monolayer double stained for F-actin and CD31 incubated without (−) or with 100 nM spike. The scale bar represents 35 μm. *C*, representative Western blot analysis of active and total RhoA, Rac1, Cdc42, and eNOS in lysates from HUVEC monolayers incubated without or with 100 nM spike in the absence or the presence of anti-α5 antibodies. *D*, ΔTEER of HUVEC monolayers incubated throughout 120 min with 100 nM spike, RBD, or RGD in the presence or the absence of antibodies against integrin α5β1 or the α5 integrin subunit. Values are means ± SD, n = 6. CD31, cluster of differentiation 31; EC, endothelial cell; eNOS, endothelial nitric oxide synthase; HUVEC, human umbilical vein endothelial cell; RGD, arginine–glycine–aspartic acid.

Besides leukocyte recruitment, β1 integrins promote vascular permeability, another critical aspect of inflammation (26). Treatment with spike, the receptor-binding domain of spike, and the RGD tripeptide induced a drop in the transendothelial electrical resistance (TEER) of the HUVEC monolayer, indicative of hyperpermeability (Fig. 5*A*). The drop was rapid, maximal at 30 min, and sustained thereafter. Furthermore, immunofluorescence showed the formation of actin stress fibers, EC retraction, and interendothelial gaps after 30 min of treatment with spike (Fig. 5*B*). Likewise, spike interfered with the peripheral distribution of cluster of differentiation 31 (CD31), an adhesion protein that maintains the junctional integrity of ECs (27) (Fig. 5*B*). These observations provided direct evidence of spike-induced increase in EC permeability.

RhoA, Rac1, and Cdc42 are Rho small guanosine triphosphatases (GTPases) that control the actin cytoskeleton and regulate EC barrier function (28). Redistribution of actin stress fibers causing EC contraction involves RhoA activation (29), whereas Rac1 and Cdc42 induce cell spreading (30). Western blot analysis of HUVEC lysates incubated with spike for 30 min showed increases in active RhoA and active Cdc42 and a decrease in active Rac1 that were blocked by the immunoneutralization of α5 (Fig. 5*C*). Furthermore, eNOS-derived NO stimulates vasopermeability (31), and spike promoted the phosphorylation/activation of eNOS in HUVECs in an α5-dependent manner (Fig. 5*C*). In contrast to the active isoforms, the total protein levels of these proteins were similar among treatments. Finally, immunoneutralization of integrin α5β1 prevented spike-induced, spike receptor–binding domain–induced, and RGD tripeptide–induced reduction in TEER (Fig. 5*D*). Taken together, these studies show that spike binding to integrin α5β1 regulates Rho GTPases and eNOS phosphorylation to promote EC hyperpermeability.

### Spike induces the hyperpermeability of ECs *in vivo*

**Figure 6:**
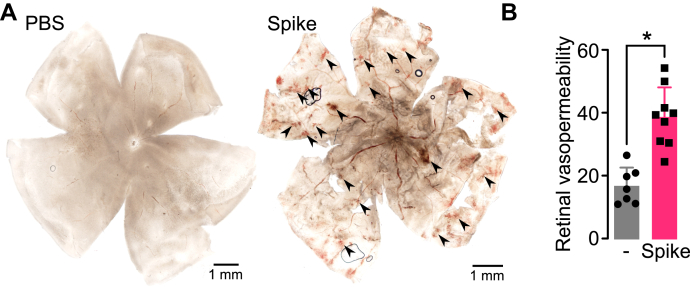
**Spike induces retinal hyperpermeability.***A*, representative images of rat flat-mounted retinas 24 h after the intravitreal injection of PBS without or with 0.5 μg of spike. Some retinal hemorrhages are indicated (*arrowheads*). *B*, retinal vasopermeability evaluated by the extravasation of Evans blue–linked albumin in rats 24 h after the intravitreal injection of PBS without or with 0.25 μg of spike. ∗*p* < 0.0001 (unpaired *t* test). Values are means ± SD, n ≥ 7.

To investigate whether the spike protein alone could promote EC hyperpermeability *in vivo*, 2 μl of vehicle (PBS) alone or containing 0.5 μg of spike was injected into the vitreous of rats, and the retinal vasculature was evaluated in flat-mounted retinas 24 h after intravitreal injection. Spike treatment caused multiple retinal hemorrhagic areas that were absent in control retinas injected with PBS (Fig. 6*A*). In other experiments, the accumulation of Evans blue–linked albumin was measured as an index of enhanced retinal vasopermeability. Spike (0.25 μg) induced a significant (greater than two-fold) increase in tracer accumulation in the retina 24 h after intravitreal injection (Fig. 6*B*). These findings show that spike disrupts the barrier function of retinal capillaries resulting in vascular leakage.

### Volociximab and ATN-161 reduce spike-induced stimulation of EC leukocyte adhesion and permeability

**Figure 7:**
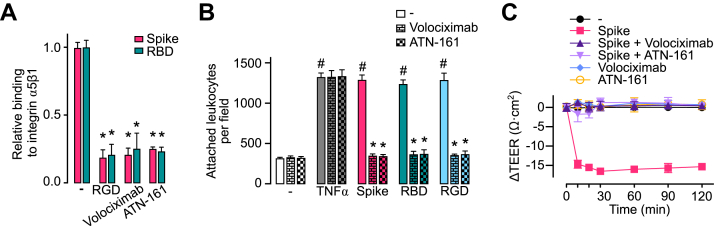
**Volociximab and ATN-161 reduce spike-induced stimulation of EC leukocyte adhesion and permeability.***A*, binding of 100 nM spike or spike receptor–binding domain (RBD) to immobilized integrin α5β1 in the absence (−) or the presence of the RGD tripeptide or the integrin α5β1 inhibitors, volociximab (5 μg ml^−1^) and ATN-161 (500 nM). *B*, leukocyte adhesion to HUVEC incubated without (−) or with 1 nM TNF⍺ or 100 nM spike, RBD, or RGD, in the absence or the presence of volociximab (5 μg ml^−1^) or ATN-161 (500 nM). #*p* < 0.001 *versus* the unstimulated control (−), ∗*p* < 0.001 *versus* the absence of integrin α5β1 inhibitors (two-way ANOVA, Tukey test). *C*, changes throughout time of the transendothelial electrical resistance (ΔTEER) of HUVEC monolayers incubated without (−) or with 100 nM spike in the presence or the absence of volociximab (5 μg ml^−1^) or ATN-161 (500 nM). ΔTEER values of HUVEC incubated only with volociximab or ATN-161 are also shown. Values are means ± SD, n = 6. EC, endothelial cell; HUVEC, human umbilical vein endothelial cell; RGD, arginine–glycine–aspartic acid; TNF⍺, tumor necrosis factor alpha.

Inhibitors of integrin α5β1 have been developed as promising therapeutics. For example, volociximab, a chimeric anti-integrin α5β1 mAb, is under clinical evaluation for the treatment of cancer (32); and preclinical studies have evaluated the efficacy of the integrin α5β1–binding peptide, ATN-161, to inhibit betacoronavirus (33) and SARS-CoV-2 virus infections (16, 17). Here, we show that both volociximab and ATN-161 block the binding of spike and the spike receptor–binding domain to α5β1 immobilized in ELISA plates (Fig. 7*A*) and prevent the leukocyte adhesion to HUVECs (Fig. 7*B*) and HUVEC hyperpermeability (Fig. 7*C*) in response to spike, spike receptor–binding domain, and RGD tripeptide.

## Discussion

Accumulating evidence has defined COVID-19 as a vascular disease (1, 2, 3, 34). Blood vessel injury causes progressive lung damage and multiorgan failure in severe COVID-19, owing to edema, intravascular coagulation, vascular inflammation, and deregulated inflammatory cell infiltration. Multiple mechanisms have been proposed for vascular dysfunction in COVID-19 (1, 2, 34); however, little is known regarding the direct action of SARS-CoV-2 on ECs (9, 11).

ACE2 is the best-established host receptor for spike (35, 36, 37), although other spike cell surface receptors have been described, that is, neuropilin-1 (38), toll-like receptors (10, 39), and RGD-binding integrins (15, 16). In particular, integrin α5β1 is an RGD-binding integrin that, upon spike binding, mediates SARS-CoV-2 entry and infection of epithelial cells and monocytes *in vitro* (16) and increases lung viral load and inflammation *in vivo* (17). Ligation of integrin α5β1 by fibronectin RGD motif activates the expression of proinflammatory genes in ECs (18), but the binding of spike to α5β1 in ECs and its impact on the EC inflammatory response has not been addressed.

**Figure 8:**
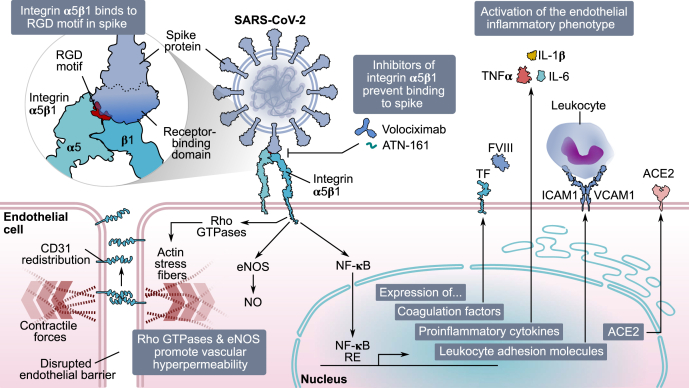
**Spike protein of SARS-CoV-2 causes endothelial dysfunction through integrinα5β1.** In EC, integrin α5β1 recognizes the RGD motif in the receptor-binding domain of SARS-CoV-2 spike protein. Spike binding activates members of the RhoA family, CD31 redistribution, actin stress fibers formation, and eNOS phosphorylation, all of which contribute to a hyperpermeable endothelium. Moreover, integrin α5β1 binding to spike activates NF-κB, which translocates into the nucleus and induces the expression of proinflammatory mediators (coagulation factors [TF and FVIII], proinflammatory cytokines [TNF⍺, IL-1β, and IL-6], leukocyte adhesion molecules [VCAM1 and ICAM1]), and ACE2. Inhibitors of integrin α5β1 (volociximab and ATN-161) block its binding to spike, preventing the activation of the vascular inflammatory phenotype. ACE2, angiotensin-converting enzyme 2; CD31, cluster of differentiation 31; EC, endothelial cell; eNOS, endothelial nitric oxide synthase; FVIII, factor VIII; ICAM1, intercellular cell adhesion molecule 1; IL-1β, interleukin 1β; IL-6, interleukin 6; RGD, arginine–glycine–aspartic acid; SARS-CoV-2, severe acute respiratory syndrome coronavirus 2; TF, tissue factor; TNF⍺, tumor necrosis factor alpha; VCAM1, vascular cell adhesion molecule 1.

In this work, we show that spike binding to integrin α5β1 activates the inflammatory program of ECs. Spike stimulated the expression of adhesion molecules ICAM1 and VCAM1 and the attachment of leukocytes to EC monolayers (Fig. 8) like TNFα, a well-known inducer of sustained EC inflammatory responses (19). Although the effect of spike on the protein levels of ICAM1 and VCAM1 was not measured, a good correlation between mRNA and protein levels has already been established for both adhesion molecules in human vascular ECs treated with different inflammatory mediators (40, 41). In addition, there is a good correlation between the dose–response curve of spike on VCAM1 and ICAM1 mRNA levels and that on leukocyte adhesion. This correlation suggests that spike upregulates the production of functional VCAM1 and ICAM1 in EC. Consistent with this notion, the intravenous administration of spike upregulated the expression of ICAM1 and VCAM1 and that of the leukocyte marker CD45 in different tissues to imply that spike stimulates leukocyte vascular attachment throughout the different vascular beds, as confirmed in retinal capillaries.

The fact that both the RGD tripeptide and the receptor-binding domain of spike elicited almost identical responses to those of spike, the RGD tripeptide itself blocked spike-binding and spike receptor–binding domain–binding to α5β1, and α5β1-neutralizing antibodies prevented the spike-induced proinflammatory effect in ECs indicated that spike, through its RGD motif in its receptor-binding domain, binds to integrin ⍺5β1 in ECs to promote inflammation (Fig. 8). Furthermore, we show that the transcription factor NF-κB is a primary contributor to α5β1 signaling in ECs in response to spike. Spike acted on ECs to stimulate IκB⍺ degradation, nuclear translocation of NF-κB, expression of adhesion molecules (ICAM1 and VCAM1), coagulation factors (TF and FVIII), proinflammatory cytokines (TNFα, IL-1β, and IL-6), and leukocyte adhesion, and treatment with the inhibitor of NF-κB and anti-α5 antibodies prevented all actions (Fig. 8). These findings are consistent with a previous study showing that NF-κB is the signaling pathway by which fibronectin ligation to α5β1 upregulates proinflammatory genes in ECs (18). Indeed, NF-κB is a predominant signaling molecule activated in ECs by proinflammatory cytokines, such as TNFα *via* an integrin-independent route (19).

Neutrophils, macrophages, and lung epithelial cells react to spike (10, 39) and to the envelope protein (42) *via* the activation of toll-like receptor–induced cytokine production, a mechanism underlying the cytokine storm observed in severe COVID-19 (25). Integrin ⍺5β1 is widely expressed in immune cells (18), and it may cooperate with toll-like receptors to boost their signaling pathways (43). However, human ECs express few or no toll-like receptors on their surface (44), implying that interactions between toll receptors and integrin ⍺5β1 do not contribute to the proinflammatory effect of spike in the endothelium. Another mechanism by which the activation of ⍺5β1–NF-κB signaling in ECs by spike can influence inflammation is the upregulation of ACE2 (Fig. 8). A previous report showed that by binding to the ACE2 receptor in ECs, spike causes the downregulation of ACE2 protein levels and the subsequent inhibition of mitochondrial function and eNOS activity leading to EC damage (9). Because ACE2 is anti-inflammatory (24), and its overexpression protects against severe COVID-19 symptoms (45), spike-induced downregulation of ACE2 would cause inflammation to thrive. Paradoxically, we found that the spike activation of ⍺5β1–NF-κB signaling in ECs increases ACE2 mRNA levels. Provided that this effect translates into more ACE2 protein, these findings suggest two contrasting possibilities: (a) upregulation of ACE2 expression is a protective mechanism against EC injury and (b) upregulation of ACE2 expression worsens viral infection of ECs by providing more SARS-CoV-2 receptor for infectivity.

Excessive vasopermeability leading to edema is another hallmark of severe COVID-19. Here, we showed that spike binding to integrin α5β1, through its RGD motif, stimulated the hyperpermeability of EC monolayers (Fig. 8). Spike-induced hyperpermeability of monolayers was mimicked by its receptor-binding domain and the RGD tripeptide and prevented by neutralizing antibodies against the α5β1 integrin and α5-integrin subunit. Furthermore, spike-induced stimulation of EC hyperpermeability occurs *in vivo* as demonstrated by the intravitreal delivery of spike resulting in multiple hemorrhagic areas and enhanced extravasation of Evans blue–linked albumin in retinal capillaries. Spike-induced disruption of the barrier function of retinal capillaries is similar to the one following intravitreal administration of vascular endothelial growth factor, a major vascular hyperpermeability factor in diabetic retinopathy, an inflammatory eye disease (46, 47).

β1 integrins are known to not only stabilize EC–cell junctions during development (48) but also promote their disruption under the context of inflammation (26). Several inflammatory agents (lipopolysaccharide, IL-1β, and thrombin) signal *via* β1 integrins to promote EC permeability and contractility *in vitro* and vascular leakage *in vivo* (49). Consistent with this observation, we showed that the spike signals through α5β1 to promote the formation of stress fibers, EC retraction, and interendothelial gap formation (Fig. 8). These processes are under the control of GTPases of the Rho family (RhoA, Rac, and Cdc42), and spike upregulated RhoA and Cdc42 and downregulated Rac1 in EC. Rho GTPases signal downstream of integrin binding to exert both positive and negative effects on EC junctions and EC matrix adhesion depending on its context. Under inflammation, RhoA associates with loss of barrier integrity, Rac1 with maintenance, and Cdc42 with barrier stabilization and recovery (50).

Consistent with the aforementioned findings, spike interfered with the peripheral distribution of CD31 observed in stable EC monolayers (Fig. 8). Because CD31 exhibits adhesive properties and is concentrated mainly at junctions between adjacent cells (27), its altered localization in ECs is consistent with increased vasopermeability. This observation agrees with a recent report showing that spike downregulates the expression of EC junctional proteins from normal and diabetic mice, reflecting the disruption of the endothelial barrier integrity (51). Finally, spike stimulated the phosphorylation/activation of eNOS in EC, and eNOS-derived NO is a principal vasorelaxant and vasopermeability factor (31, 52) (Fig. 8).

Altogether, we have provided *in vitro* and *in vivo* evidence to show that by binding to α5β1, spike changes the EC phenotype to promote vascular inflammation (Fig. 8). Upon spike-mediated α5β1 activation, ECs lose their ability to control permeability or quiesce leukocytes, both of which are characteristics of EC dysfunction (19). These findings provide insights into the mechanisms making COVID-19 a vascular disease and encourage the development of therapeutic approaches that directly focus on α5β1-mediated vascular changes.

Because the spike protein is the main immunogen in COVID-19 vaccines, the notion that immunization with spike could cause EC dysfunction should be discussed. In contrast to the spike protein levels (∼30 ng ml^−1^) found in severe COVID-19 (53), the circulating levels of the spike protein after an mRNA vaccine are minute (∼30 pg ml^−1^) (54), much lower than the EC_50_ value of spike determined in this study (∼300 ng ml^−1^). Furthermore, most of the spike remains attached to the cell surface and does not disperse much from the injection site (54). In addition, spike is no longer detected in circulation after the second dose of vaccine, presumably because antibodies generated by the first immunization quickly and effectively remove the minute amounts of spike reaching the circulation (54). Therefore, vaccination should help prevent, and not worsen, EC damage in COVID-19.

Finally, we showed that two inhibitors of α5β1, volociximab and ATN-161, developed as promising therapeutics, blocked spike-induced leukocyte adhesion and hyperpermeability of EC. Indeed, ATN-161 has already been shown to inhibit SARS-CoV-2 virus infection *in vivo* (17). Our findings, add ECs as a target for these drugs to reduce vascular inflammation in COVID-19, suggest the therapeutic potential of agents commonly used to treat vascular diseases and provide tools for guiding research into the molecular mechanisms mediating the pathophysiology of COVID-19.

## Experimental procedures

### Reagents

Recombinant SARS-CoV-2-His-tagged spike (active trimer) (catalog no. 10549-CV) was purchased from Bio-Techne. Recombinant receptor-binding domain of spike (amino acids 319–541) flanked by the signal peptide (amino acids 1–14) and containing a 6×His tag was produced as previously reported (55). RGD tripeptide, the NF-κB activation inhibitor BAY 11-7085, and ATN-161 were purchased from Sigma–Aldrich, and human recombinant TNFα was from R&D Systems. Purified human integrin α5β1 and anti-α5 (MAB1956Z) and anti-α5β1 (MAB1999) antibodies were from Merck. Volociximab was from Novus Biologicals.

### Cell culture

HUVECs were obtained as described (56). HUVEC were maintained in F12K media supplemented with 20% fetal bovine serum (FBS), 100 μg ml^−1^ heparin (Sigma–Aldrich), 25 μg ml^−1^ EC growth supplement, and 100 U ml^−1^ penicillin–streptomycin.

### Leukocyte adhesion assay

HUVECs were seeded on a 96-well plate and grown to confluency. HUVEC monolayers were treated for 16 h with TNFα, spike, the spike receptor–binding domain, and the RGD tripeptide alone or in combination with anti-α5β1 antibodies (5 μg ml^−1^), anti-α5 antibodies (5 μg ml^−1^), volociximab (5 μg ml^−1^), or ATN-161 (500 nM) in 20% FBS F12K without heparin or EC growth supplement. For inhibition of NF-κB, cells were pretreated for 30 min with 5 μM of BAY 11-7085. Leukocytes were prepared from whole blood collected into heparin tubes. Briefly, blood was centrifuged (300*g* for 5 min), and the plasma layer was discarded. The remaining cell pack (∼5 ml) was diluted in 45 ml of red blood cell lysis buffer containing 150 mM of NH_4_Cl, 10 mM NaHCO_3_, and 1.3 mM EDTA (disodium) and gently rotated for 10 min at room temperature (RT). Leukocytes were collected by centrifugation (300*g* for 5 min), the pellet was washed with cold PBS followed by centrifugation (300*g* for 5 min), and resuspended into 5 ml of warm PBS containing 5 μg ml^−1^ Hoechst 33342 (Thermo Fisher Scientific) to stain live leukocytes. Leukocytes were incubated in darkness for 30 min, washed with PBS three times by centrifugation (200*g* for 5 min each), resuspended into supplemented culture media, and taken to 10^6^ cells ml^−1^. After 16 h of treatment, the medium of HUVEC monolayers was replaced with 100 μl of medium containing Hoechst-stained leukocytes (∼100,000 leukocytes per well), and cells incubated for 1 h at 37 °C. HUVEC monolayers were washed three times with warm PBS, and images were obtained in an inverted fluorescent microscope (Olympus IX51) and quantified using the CellProfiler software from the Broad Institute (57).

### ELISA

A 96-well ELISA microplate was coated overnight at 4 °C with 25 ng per well of human integrin α5β1 diluted in PBS and blocked for 1 h at RT with 5% w/v nonfat dry milk in 0.1% PBS with Tween-20 (PBST). After blocking, microplates were washed three times with PBST, and different concentrations of spike or spike receptor–binding domain were added. Anti-α5β1 antibodies (5 μg ml^−1^), the RGD tripeptide (500 nM), ATN-161 (500 nM), or volociximab (5 μg ml^−1^) were added together with 100 nM of spike or spike receptor–binding domain. Dilutions were in 0.2 mg ml^−1^ bovine serum albumin (BSA)–PBST. Microplates were then incubated for 1 h at RT, washed three times with PBST, incubated for 1 h at RT with 1:1000 of antispike antibody (rabbit mAb; Sino Biological) diluted in blocking buffer, washed (three times in PBST), and incubated (1 h at RT) with horseradish peroxidase–labeled goat anti-rabbit secondary antibodies (Thermo Fisher Scientific) diluted 1:2500 in 50% blocking buffer. After a three-wash step, the microplates were incubated with 100 μl of an o-phenylenediamine dihydrochloride substrate tablet diluted in 0.03% H_2_O_2_ citrate buffer for 30 min in darkness. The reaction was stopped with 50 μl of 3 M HCl, and the absorbance was measured at 490 nm.

### NF-κB nuclear translocation analysis

HUVECs were seeded on 18 mm coverslips coated with fibronectin (1 μg cm^−1^) and placed in a 12-well plate. Cells were grown in complete media to 80% confluence, and on the day of the assay, the medium was replaced with 0.5% FBS–F12K. The NF-κB activation inhibitor, BAY 11-7085 (5 μM), or anti-α5 antibodies (5 μg ml^−1^) were added 30 min before a 30 min incubation with spike (100 nM) or TNFα (1 nM). Cells were washed, fixed in 4% paraformaldehyde (PFA) for 30 min at RT, permeabilized for 30 min with 0.5% Tx-100-PBS, blocked with 0.05% Tx-100, 1% BSA, 5% normal goat serum–PBS at RT for 1 h, and incubated overnight at 4 °C in a humid chamber with 1:200 mouse monoclonal anti–NF-κB p65 antibody in 0.1% Tx-100, 1% BSA–PBS. Cells were washed and incubated under darkness at RT with a goat antimouse secondary antibody (1:500 dilution; Alexa Fluor 488; Abcam) in 0.1% Tx-100 and 1% BSA–PBS for 2 h. Nuclear DNA was counterstained with 5 μg ml^−1^ Hoechst 33342 (Sigma–Aldrich), and the coverslips were washed and mounted with Vectashield mounting medium (Vector laboratories) and observed under fluorescence microscopy (Olympus IX51).

### Quantitative PCR of HUVECs

HUVECs grown to 80% confluency on 6-well plates were incubated under starving conditions (0.5% FBS and F12K medium) for 30 min with the NF-κB activation inhibitor, BAY 11-7085 (5 μM) or anti-α5 antibodies (5 μg ml^−1^) followed by a 4 h incubation with 100 nM spike. RNA was isolated using TRIzol reagent (Invitrogen), retrotranscribed with the high-capacity complementary DNA (cDNA) reverse transcription kit (Applied Biosystems), and quantified using Maxima SYBR Green qPCR Master Mix (Thermo Fisher Scientific) in a final reaction of 10 μl containing 20 ng of cDNA, and 0.5 μM of each of the following human primers: ICAM1: forward (5′-gtgac cgtgaatgtgctctc-3′) and reverse (5′-cctgcagtgcccattatgac-3′); VCAM1: forward (5′-gcactgggttgactttcagg-3′) and reverse (5′-aacatctccgtaccatgcca-3′); F3/TF: forward (5′-tgtatgggccaggagaaagg-3′) and reverse (5′-cccactcctgcctttctaca-3′); F8/FVIII: forward (5′-aaagactcacattgatggcc-3′) and reverse (5′-tctggatttt gtgcatctgg-3′); TNF⍺: forward (5′-accacttcgaaacctgggat-3′) and reverse (5′-tcttctcaagtcctgcagca-3′); IL-1β: forward (5′-ggagaatgacctgagcacct-3′) and reverse (5′-ggaggtggagagctttcagt-3′); IL-6: forward (5′-cctgatccagttcctgcaga-3′) and reverse (5′-ctacatttgccgaagagccc-3′); chemokine (C-X-C motif) ligand 8/IL-8: forward (5′-gcagagggttgtggagaagt-3′) and reverse (5′-accctacaacagacccacac-3′); chemokine (C-C motif) ligand 2: forward (5′-gcaagtgtcccaaagaagct-3′) and reverse (5′-gctgcagattctt gggttgt-3′); ACE2: forward (5′-ccgaaatacgtggaactcatcaa-3′) and reverse (5′-cacgagtcccctgcatctaca-3′), and GAPDH: forward (5′-gaaggtcggagtcaacggatt-3′) and reverse (5′-tgacggtgccatggaatttg-3′). The amplification conditions were 10 s at 95 °C, 30 s at each primer pair–specific annealing temperature, and 30 s at 72 °C for 40 cycles. The mRNA expression levels were calculated by the 2^−ΔΔCT^ method normalized to the human GAPDH transcript.

### Vasopermeability assay

HUVECs were grown to confluence on a 6.5 mm transwell with a 0.4 μm pore coated with 1 μg cm^−1^ fibronectin (Thermo Fisher Scientific). TEER was measured using the epithelial EVOM^2^ Volt/Ohm meter (World Precision Instruments). The assay was performed once the TEER measurement was stable for at least 2 days. Cells were pretreated for 5 min with anti-α5 antibodies (5 μg ml^−1^), anti-α5β1 antibodies (5 μg ml^−1^), volociximab (5 μg ml^−1^), or 500 nM ATN-161 followed by the treatment with 100 nM spike, spike receptor–binding domain, or the RGD tripeptide. TEER measurements were made over 120 min.

### Fluorescence cytochemistry for CD31 and F-actin

HUVECs were seeded on 18 mm coverslips coated with fibronectin (1 μg cm^−1^) and placed in a 12-well plate. Cells were grown to confluence, and once the monolayer was completely formed, cells were starved (0.5% FBS) for 1 h before the addition of 100 nM of spike for 30 min. Cells were washed, fixed with 4% PFA for 30 min, permeabilized with 0.5% Tx-100-PBS for 30 min, blocked with 0.1% Tx-100, 1% BSA, 5% normal goat serum–PBS for 1 h at RT, and incubated overnight at 4 °C in a humid chamber with 1:100 anti-CD31 antibody (Abcam) in 0.1% Tx-100 and 1% BSA–PBS. Cells were washed and incubated in darkness with goat antimouse secondary antibodies (1:500 dilution; Alexa Fluor 488) in 0.1% Tx-100 and 1% BSA–PBS for 2 h. Cells were then washed with PBS, and 300 μl of 160 nM rhodamine–phalloidin (Thermo Fisher Scientific) was added to each well for 1 h in darkness, followed by a PBS wash and nuclear DNA counterstaining with 5 μg ml^−1^ Hoechst 33342 (Sigma–Aldrich). Finally, the coverslips were washed, mounted, and observed under fluorescence microscopy.

### RhoA–Rac1–Cdc42 analysis

HUVECs were seeded on a 6-well plate and grown in complete media. Eighty percent confluent cells were then starved for 3 h (0.5% FBS) and treated or not for 30 min with the anti-α5 antibodies (5 μg ml^−1^) followed by the addition of 100 nM spike for an incubation of 30 min. Cells were washed twice with ice-cold PBS, and the RhoA–Rac1–Cdc42 activation was evaluated using the RhoA–Rac1–Cdc42 combo activation assay kit (Abcam) according to the manufacturer's instructions. The kit is based on the binding of the active form of RhoA to the Rho-binding domain of rhotekin and of active Rac1 or Cdc42 to the p21-binding domain of p21-activated protein kinase 1. Both, rhotekin and p21-activated protein kinase 11 binding domains are coupled to agarose beads, and the isolation and detection of the active forms of RhoA, Rac1, and Cdc42 were done under conventional bead precipitation and Western blot protocols using goat antimouse alkaline phosphatase secondary antibodies (Jackson ImmunoResearch; 1:5000 dilution) and a colorimetric detection kit (Bio-Rad). Total levels of RhoA, Rac1, and Cdc42 were measured by Western blot in the absence of the isolation step.

### Western blot

HUVECs were seeded on a 6-well plate and grown in complete media to reach an 80% confluency. On the day of the assay, HUVECs were starved (0.5% FBS) for 3 h and treated or not for 30 min with the anti-α5 antibody (5 μg ml^−1^) followed by the addition of 100 nM spike for an incubation of 30 min. Cells were washed twice with cold Tris-buffered saline, scraped with 200 μl radio immunoprecipitation assay buffer supplemented with 1:100 halt protease–phosphatase inhibitor cocktail (Thermo Fisher Scientific) and 5 mM of EDTA, centrifuged (10,000*g*, 4 °C for 10 min), and supernatants aliquoted and stored (−70 °C) for Western blot analysis. Forty-five micrograms of protein were resolved in SDS-PAGE and blotted with anti–phospho-eNOS (Ser1177) antibodies (Cell Signaling; 1:250 dilution) or anti–NF-κB inhibitor-α (IκBα) (1:200), followed by incubation with goat anti-rabbit horseradish peroxidase secondary antibodies (Jackson ImmunoResearch; 1:5000 dilution). Immunoblots were developed using the SuperSignal West Pico PLUS chemiluminescent substrate kit (Thermo Fisher Scientific) and the FluorChem E imager and gel documenter system (ProteinSimple). Membranes were reblotted using antibodies against total eNOS (Cell Signaling; 1:500 dilution) or anti-β-tubulin antibodies (Abcam; 1:1000 dilution), goat anti-rabbit alkaline phosphatase secondary antibodies (Jackson ImmunoResearch; 1:5000 dilution), and a colorimetric detection kit (Bio-Rad).

### Animals and ethical statement

Animals were maintained under standard laboratory conditions (24 °C, 12 h/12 h light/dark cycle) with free access to food and water. Experiments were approved by the Bioethics Committee of the Institute of Neurobiology of the National University of Mexico (Universidad Nacional Autónoma de México [UNAM]) according to the US National Research Council's Guide for the Care and Use of Laboratory Animals (Eighth Edition, National Academy Press).

### *In vivo* vascular inflammation

Female C57BL6 mice (8–12 weeks old) were i.v. injected with 2.7 μg of spike in 50 μl of PBS. After 2 h, animals were euthanized by cervical dislocation and intracardially perfused with at least 10 ml of PBS to remove the blood and nonadhered leukocytes. Eyes, lung, liver, and kidney tissues were placed immediately in TRIzol reagent, retrotranscribed with the high-capacity cDNA reverse transcription kit (Applied Biosystems), and transcription products quantified using Maxima SYBR Green qPCR Master Mix (Thermo Fisher Scientific) in a final reaction of 10 μl containing 20 ng of cDNA, and 0.5 μM of each of the following mouse primers: ICAM1: forward (5′-gctgggattcacctcaagaa-3′) and reverse (5′-tggggacaccttttagcatc-3′); VCAM1: forward (5′-attgggagagacaaagcaga-3′) and reverse (5′-gaaaaagaaggggagtcaca-3′); TNF⍺: forward (5′-catcttctcaaaattcgagtgacaa-3′) and reverse (5′-tgggagtagacaaggtacaaccc-3′); IL-1β: forward (5′-gttgattcaaggggacatta-3′) and reverse (5′-agcttcaatgaaagacctca-3′); IL-6: forward (5′-ga ggataccactcccaacagacc-3′) and reverse (5′-aagtgcatcatcgttgttcataca-3′); CD45: forward (5′-tatcgcggtgtaaaactcgtca-3′) and reverse (5′-gctcaggccaagagactaacgt-3′); and GAPDH: forward (5′-gaaggtcggtgtgaacggatt-3′) and reverse (5′-tgactgtgccgttgaatttg-3′). The amplification conditions were 10 s at 95 °C, 30 s at each primer pair–specific annealing temperature, and 30 s at 72 °C for 35 cycles. The mRNA expression levels were calculated by the 2^−ΔΔCT^ method normalized to the mouse GAPDH transcript.

### Leukocyte adhesion to retinal vessels

The method previously described was used (58). Briefly, female C57BL6 mice (8–12 weeks old) were i.v. injected with 2.7 μg of spike in 50 μl of PBS. After 4 h, animals were euthanized by cervical dislocation, intracardially perfused with 10 ml of PBS, to wash nonadhered leukocytes, followed by perfusion with 5 ml fluorescent isothiocyanate–labeled concanavalin A (ConA) lectin (40 μg ml^−1^ in PBS, pH 7.4; Invitrogen), to label adherent leukocytes and EC. Residual unbound ConA was then removed by perfusion with 10 ml of PBS. Eyes were fixed in 4% PFA for 10 min, retinas dissected, maintained in cold methanol for at least 20 min, washed, permeabilized, and blocked with PBS containing 1% Triton X-100, 0.4% BSA, and 10% normal goat serum for 48 h at 4 °C. Retinas were then immunostained for 48 h at 4 °C with a 1:200 dilution of anti-CD45 mAb (Santa Cruz), washed, and labeled overnight with a 1:500 dilution of Alexa Fluor 555 goat antirat secondary antibody (Invitrogen). Finally, retinas were washed, flat mounted, and cover slipped using Vectashield H-1000 (Vector Laboratories, Inc). Retinal vessels and intravascular ConA+ and CD45+ cells, identified as leukocytes, were digitized under a fluorescence microscope.

### Retinal vasopermeability assays

Male Wistar rats (250–300 g) anesthetized with 1 μl g^−1^ of a mix of 60% ketamine and 40% xylazine were intravitreally injected with 2 μl of vehicle (PBS) containing 0.5 μg or 0.25 μg of spike for flat-mounted retina or Evans blue method evaluation of retinal vasopermeability, respectively. Vasopermeability assays were carried out 24 h after intravitreal injections. Retinas were flat mounted, fixed for 15 min in 4% PFA at RT, washed, mounted on glass slides with 50% glycerol (Sigma–Aldrich), and observed under light-field microscopy. The Evans blue method was as previously described (59). Briefly, anesthetized rats were injected intrajugularly with 45 mg kg^−1^ of the Evans blue tracer (Sigma–Aldrich), and after 2 h, 1 ml of blood was obtained from the heart to quantify plasma Evans blue concentration. Rats were then perfused with 200 ml PBS (pH 3.5 at 37 °C) at 70 ml min^−1^ *via* left ventricle, and the retinas were dissected, vacuum dried for 4 h, and weighted. The Evans blue tracer was extracted in 250 μl of formamide (Sigma–Aldrich) at 72 °C for 18 h. Absorbance was measured in the supernatant at 620 nm. The tracer concentration in retinal extracts was calculated through the interpolation of the absorbance at 620 nm of the supernatants to those of a standard curve of Evans blue in formamide. Values were normalized by the plasma concentration and to the retina–body weight ratio.

## Data availability

All data generated or analyzed during this study are included in this article.

## Conflict of interest

The authors declare that they have no conflicts of interest with the contents of this article.
